# Morally relevant features warranting ethical oversight in human stem cell-based embryo models

**DOI:** 10.1038/s41556-026-01881-4

**Published:** 2026-04-08

**Authors:** Naomi Moris, Alfonso Martinez Arias, Martin Pera, Nicolas Rivron, Karen Sermon, Nienke de Graeff

**Affiliations:** 1 The Francis Crick Institute, 1 Midland Way, London, NW1 1AT; 2 Systems Bioengineering, DCEXS, Universidad Pompeu Fabra, Doctor Aiguader 88 ICREA ( Institució Catalana de Recerca i Estudis Avançats), Barcelona, Spain; 3 The Jackson Laboratory, Bar Harbor, ME, USA; 4 Institute of Molecular Biotechnology of the Austrian Academy of Sciences (IMBA), Vienna Biocenter (VBC), 1030 Vienna, Austria; 5 Research Group Genetics, Reproduction and Development (GRAD), Vrije Universiteit Brussel, Brussels, Belgium; 6 Department of Medical Ethics & Health Law, Leiden University Medical Center, Leiden University, Leiden, the Netherlands; 7 The Novo Nordisk Foundation Center for Stem Cell Medicine (reNEW), Leiden Node, Leiden, The Netherlands

## Abstract

Stem cell-based embryo models (SCBEMs) provide innovative ways to explore the principles of human development but also pose new challenges for regulation. Recent guidelines have argued for case-by-case evaluation of SCBEM research, but it is still unclear what exactly such oversight should consider. Here, we argue that effective review requires identification of a set of attributes by which to evaluate research proposals. To define these, the underlying ethical values and morally relevant biological features present in embryo models must be identified, to enable the practical implementation of oversight. We propose that developmental stage, tissue/organ integrity, fetal potential and the capacity to form neural circuits should be used together for evaluation purposes. We also highlight the importance of the wider context, including the proposed uses of embryo models, public perception and individual researcher responsibilities and thus provide a general framework for regulatory consideration of human embryo model research.

The rapidly advancing field of stem cell-based embryo models (SCBEMs) provides opportunities to investigate human-specific developmental processes. However, it also raises significant ethical issues that are complicated by the fact that embryo models are incredibly diverse, technical approaches advance quickly, and our current knowledge of the developmental potential of SCBEMs is limited. As a result, it is difficult to categorise SCBEMs for regulation, and scientific advances often blur the boundaries proposed by regulatory frameworks. Clear, timely guidance is needed to facilitate consistent decision-making by local and international regulatory bodies, such as Stem Cell Research Oversight committees (SCROs).

Recently, the International Society for Stem Cell Research (ISSCR) updated their guidelines, suggesting that all SCBEM research be subject to review by a local oversight committee[1], with the exception of only the most simple models, such as 2D micropatterns and organoids. Previously, SCBEMs were classified into ‘integrated’ or ‘non-integrated’ models, depending on the presence or absence of extraembryonic tissues - originally intended as a proxy for their capacity to develop further - with only integrated models requiring review[2]. However, recent research highlighted numerous limitations to this distinction, including both technical and ethical considerations. In particular, concerns emerged[3, 4] that extraembryonic tissues are not strictly necessary for in vitro entities to develop morally relevant features. Because of this, the UK Code of Practice[5] and Nuffield Council of Bioethics report [6] both recommended case-by-case evaluation in lieu of using the integrated/non-integrated distinction, as was then also adopted in the updated ISSCR guidelines[7]. This highlights the need for regulatory criteria to be aligned with underlying morally relevant features, and demonstrates the challenge and necessity of defining categories for SCBEM oversight that remain consistent with scientific insights.

However, it is not yet clear exactly how research should be ethically evaluated on a case-by-case basis[4, 5, 7]. The UK Code of Practice[5] proposed to have a degree of oversight that is *“proportionate to the complexity of the SCBEM and proportionate for each individual project”*, and the 2025 ISSCR guidelines[7] recommend that oversight should be *“proportionate to the complexity of the model”*. However, the metrics and ethical principles by which such complexity and proportionality should be assessed remain unclear, making implementation challenging and risking inconsistent application by different regulatory committees.

To address this gap, this Perspective aims to elucidate ethical values and principles relevant to SCBEM research, and then identify related biological ‘morally relevant features’ that might be present in SCBEMs. We suggest that researchers should justify the scientific rationale for using a particular SCBEM based on the presence of these morally relevant features, alongside the anticipated impacts (i.e., benefits and risks) of their investigations. Here, we focus solely on human SCBEMs but it is possible that some non-human SCBEMs, e. g. of non-human primates, might also raise their own ethical considerations. While not necessarily presenting an exhaustive list of morally relevant features, we hope this may serve as an initial basis to devise more detailed ethical oversight procedures and so guide consistent decision-making by scientists, regulators, and law- and policymakers.

## Embryo versus SCBEM research

In devising regulatory principles to apply to SCBEM research, an obvious first step is to consider the regulation of embryos derived through fertilization for guidance. In many ways, the regulation and legislation of embryo research have been a resounding success; they have been adopted internationally, provided robust boundaries and enabled scientific exploration, while balancing public trust in research^[8]^. In particular, the benefit of a fixed limit to human embryo research, the so-called ‘14 Day rule’, is its simplicity: as Warnock herself said, “everyone can count to 14”[9]. Although there have been recent calls to extend this limit to 28 days[10], the chronological limit to embryo research has been crucial for its broad public support. This straightforward boundary provides a pragmatic cut-off point to regulate research, by preventing all subsequent progression, without specifying precisely which features or stages it wishes to avoid. As such, the Day 14 rule promotes public trust by imposing a limit to research on embryos before their development raises significant objections, allowing scientists to undertake research with potential benefits.

However, it is increasingly clear that the same principle of a chronological, day-based limit cannot be applied to all SCBEMs because models can skip stages of development (e.g., fertilisation, implantation), they do not necessarily develop at a predictable pace, and there are partial models that contain certain some cells and tissues but not all of those found in the embryo at equivalent stages[11]. Therefore, the implementation of a solely time-based limit is not sufficient for SCBEM regulation[12]. Thus, we instead need to identify the ethical values and biological features that are relevant to SCBEM research, in order to ensure appropriate ethical oversight.

## What we want to protect

As a basic starting point, we propose that there are broad general values we might want to consider with respect to either embryo or SCBEM research: (i) intrinsic value, (ii) extrinsic value, and (iii) public trust in science[13, 14].

Intrinsic value relates to the moral status or value that the entity itself has, which may be based on its features or properties, such as the ability to feel pain, to be aware of surroundings, or to be self-conscious[14, 15]. However, as discussed further below, the boundaries surrounding these properties are not always clear.

Extrinsic value, by contrast, relates the value of an entity to something else to which it is related. For instance, some may attribute value to human embryos because of the intention behind their creation, such as their societal role in family building[16]. Likewise, for some people an embryo may hold symbolic value, not necessarily as a person or individual in its own right, but because of what it represents [17][18].

The third value is public trust in science, as regulation is often essentially a form of ‘social contract’ that must balance wide-ranging points of view, objectives and perceptions[8]. As such, any regulation must consider and weigh the diverse values of members of society [19].

## Morally relevant features

Having outlined the general values to consider in SCBEM research, a major challenge is to identify the biological features that are morally relevant. While an exact one-to-one alignment between biological features and moral relevance would be ideal, it is difficult to identify which features attribute moral value[15] (i.e., what exactly makes a human embryo hold moral value), and this is further complicated by limitations in our scientific understanding of underlying processes (e.g., exactly when or how pain perception emerges). Additionally, certain biological characteristics might not necessarily confer intrinsic moral value but still raise wider concerns, for instance among members of the public. To address this challenge, we propose here a set of morally relevant features that we feel should be part of the holistic evaluation of research; these include (i) developmental stage, (ii) integration of organ systems, (iii) fetal potential, (iv) neuronal circuit-forming capacity and (v) features of public concern.

These morally relevant features are not intended to serve as simplistic, binary “cut-off” points (what is allowed versus not allowed), nor to imply a single linear scale (a given model being more concerning than another), but as a multifactorial and therefore multidimensional evaluation. We discuss these features in more detail below.

### Developmental stage

Human development involves dynamic transitions along a well-defined continuum, and progression along this trajectory is a critical feature of both human embryos and SCBEMs.

Ethically, different opinions exist with regard to whether and if so, how the moral value of an embryo changes over developmental time[18]. We have previously argued that moral consideration increases during development in step with the emergence of certain structures that might confer moral value[20]. Given that current ethical regulation of human embryos relies strongly on the termination of experiments at a defined point in order to prevent subsequent stages of development, it seems logical to apply the same principle of a ‘set end point’ also to SCBEMs.

While some SCBEMs do not adhere to canonical developmental timelines, making a simplistic chronological regulation impractical[5, 6], the explicit purpose of SCBEMs is to provide insight into embryonic processes[21]. Because of this, researchers are likely aware of which ‘developmental window’ they intend their model to replicate in vitro. Correspondingly, we suggest that researchers should propose to investigate within a given developmental window, with a defined endpoint, and no further (e.g., the process of somitogenesis until the epithelial-to-mesenchymal transition of somitic mesoderm). If researchers wanted to proceed beyond this, they would then need to seek additional review, with an updated developmental window proposed and associated scientific justification. Any such developmental windows need not merely be time-based (i.e., number of days/weeks in culture) but could be linked instead to the emergence of certain features, cell types or embryonic equivalent stages, such as Carnegie Stages[6]. These defined endpoints should be proposed by the researchers themselves at the point of application, alongside a proposal outlining the frequency and methods to determine whether this endpoint has been reached, allowing the committee to decide whether this suggestion was reasonable, sufficient and ethically justified.

### Integration of organ systems

Comparisons are often drawn between SCBEMs and organoids, since both are stem cell-based, three-dimensional cellular ensembles with self-organising properties that recapitulate dynamical biological processes *in vitro*. However, there is a major distinction between organoids and SCBEMs: organoids model a single organ or tissue, while SCBEMs co-develop multiple tissues and replicate the developmental dynamics of the embryo. This distinction becomes blurred as SCBEMs transition beyond the early stages and into organogenesis, and as organoids are becoming increasingly complex or are grown as ‘assembloids’ or ‘multi-organoids’[22] We argue multilineage SCBEMs comprising several organ systems that are all integrated into a single entity, warrant additional moral scrutiny compared to a set of disconnected, single organoids, for two main reasons.

Firstly, biological systems often show emergent features when components are integrated. For instance, while a simple collection of retinal and cortical neuronal cells might have little moral value individually, when they are integrated as a circuit, the combined entity may have an increased moral value because it is now capable of morally relevant complex processes – in this case, integration of sensory input into higher processing functions – beyond the sum of its parts. Similarly, a model containing functionally beating cardiac tissue alongside the full system of blood and vascularisation could become capable of more prolonged development (thus increasing its potential) than if any of those components were present individually.

The second reason is linked to model composition. A SCBEM that only partially models the fetal body plan (eg somitoids[23], axioloids[24] or trunk-like structures[25]) are likely to be less morally concerning than those that recapitulate the entirety of the fetal bodyplan organisation (see for instance, mouse models with fairly complete bodyplans[26, 27], if these were translated into human equivalents). This is particularly pertinent due to uncertainties about the minimal complexity required to provide moral status[15] and the possible potential to develop further (see below).

Correspondingly, SCBEMs that integrate many or all organ systems warrant additional oversight to evaluate their possible emergent features, potential to develop further, and moral status. Notably, it is not necessarily the case that SCBEMs with more organ components are always more ethically concerning than SCBEMs with fewer organ components, since many factors are important, including the number of tissues, the type of tissues, how they relate to one another, and how they are organised.

We propose that researchers should explain which tissues/organs might be expected to emerge during their SCBEM research (as well as any that might not) with a scientific justification. They should also describe whether any action will be taken to minimise or prevent the integration and interaction of organ systems that are present. Models that do not recapitulate the entirety of the bodyplan, and that minimise the integration of organ systems, should be favoured wherever the scientific motivation allows a more minimal model to be used. This would thus adhere to the principles of proportionality (ensuring that the benefits outweigh the risks) and the least infringement principle (by using the model with fewest or minimal morally relevant features)[28].

### Fetal potential

Part of the reason that increasing model organ integration may be of ethical relevance is owing to its link with a SCBEM’s potential to form a fetus, or even live offspring. Some proposals have focused on the notion of ‘developmental potential’[11, 29, 30] to distinguish between models based on their future capacity. Various arguments have been made regarding the use of ‘developmental potential’ in ethically evaluating biological systems[30, 31].There are many facets to this especially since the concept can be understood as a matter of possibility, probability or predisposition – the last of which further distinguishes between active potential (the entity is intrinsically able to fulfil this potential under supportive circumstances) or passive potential (the entity is only able to fulfil this potential after an external intervention to acquire this capacity). Depending on the particular understanding at hand, the concept of ‘developmental potential’ can become too broad as it implies any capacity to achieve dynamic developmental outcomes which may equally apply to stem cells in adherent culture. For the purposes of evaluating SCBEM research, we propose that it could be relevant to distinguish between entities that do and do not have the *potential to develop to fetal stages*.

The feature of fetal potential should be considered as a combined function of the intrinsic conditions of the SCBEM to develop into a fetal-stage entity (because of integration of multiple organ systems and advanced developmental stage, for example), alongside the extrinsic conditions (such as the experimental design or bioengineering approach) that might actually enable prolonged development and the realization of morally relevant features. This inclusion not only of the model characteristics but of the experimental design and application should allow fetal potential to be evaluated not just as latent potential but also including evaluation of practices that move towards the realisation or actuation of the potential to develop to fetal stages.

This will ensure that simplistic models cultured for short periods of time require less oversight than those with the potential to reach highly advanced stages. It also implies that SCBEMs that do not have the necessary intrinsic conditions, yet for which there is a clear intention to supply the missing components through external conditions, are regulated as stringently as those that inherently possess such capacities.

### Capacity to form functional neural circuits

It is largely agreed that a human entity capable of feeling pain and being conscious should be considered to have full moral status [15]. What is much less clear is whether and to what extent entities with partial, incomplete or curtailed aspects of these features should be attributed moral status as well as when, biologically, the anatomical features enabling this capacity can be said to be functionally present.

Scientific arguments regarding the point at which a fetus is considered to be sentient, cover a wide range of weeks^24^. For many, the presence of the cortex and connection of the peripheral nervous system through the spinal cord and thalamus is the key indicator that pain perception may be possible, and this is likely to occur at 22-24 weeks of fetal development^25–27^. Others have argued that since the first neural projections from the thalamus to the subcortical plate happen at 12-18 weeks^28^, this may be a more suitable cut-off point for possible pain perception based on neurological morphology^29^. More basic touch sensitivity is already present around eight weeks of development[32]. Accurately determining the biological features associated with pain perception is complicated by whether pain is defined as a conscious perception or a physical response. Moreover, the emergence of consciousness in human development is even less well defined.

When considering SCBEMs, an added difficulty is in identifying the minimal biological components that are necessary for such properties. Without a solid biological understanding of the mechanisms underlying complex traits such as consciousness, sentience or sensory avoidance, it remains difficult to predict whether an advanced SCBEM might exhibit higher-order neural characteristics.

Therefore, we argue that, here, we should follow the principle of precaution. Any experimental design involving new SCBEMs should take active steps to ensure that it stops short of an entity that could cross the boundaries of sentience or consciousness, unless very strong reasoning is provided to support the need for such experiments. This might mean considering whether the model will develop central neural processing capacity or mature states of neuromuscular circuitry. Such considerations would ensure that SCBEMs do not develop features that might have the capacity to cross this threshold of developing neural processing. Researchers should therefore state whether they expect their research to lead to entities with the capacity to form neural circuits and why (or why not), including active means by which they will ensure that their experiments stop well short of pain perception or consciousness.

### Features of public concern

Additionally, it is important to consider the context of research within society. Key to ensuring public trust in SCBEM research is to engage in public dialogue to obtain a comprehensive view of how the public perceive research, as has been completed in the UK[33] and the Netherlands[34]. These dialogues found that while there was overall excitement about opportunities presented by SCBEM research, many participants wanted to see clearer regulation and increased public involvement in governance discussions. Although opinions are likely to vary significantly within and across different segments of society and populations, public concerns might arise when models develop features such as beating cardiac structures, appendages (hands/feet and digits) or the rudiments of facial structure, or other defining and identifying features of human anatomy [33]. While these structures do not necessarily possess any intrinsic moral value themselves, such biological features may still evoke a response that should be identified, further discussed and evaluated [35].

Wherever possible, researchers should engage in dialogue events including the public, and funding sources should be made available for this purpose, particularly to target otherwise underrepresented segments of society. When doing so, researchers should consider how they aim to responsibly manage communications and actively engage with the public to discuss potential concerns in a responsive and dynamic two-way manner. Oversight committees should also strive to include the public and lay members within their composition, to include this voice as part of the evaluation of research.

It is important to note that we are not arguing that public considerations should be used to categorise models, nor to define acceptable and unacceptable boundaries for research. We instead suggest that public perception should be used as part of an assessment of the wider context of a SCBEM, and that the specific models for which morally relevant features have been identified through public engagement should be subjected to additional moral scrutiny to ensure that researchers are minimising any risks and addressing identified ethical concerns.

## Responsibilities of researchers and regulators

While the categories proposed here aim to identify morally relevant features of SCBEMs, we stress that in every scenario, a clearly articulated scientific justification for all research is required[36], as is due diligence to balance possible risks of the research against its likely benefits. Some research proposals that incorporate many or all of these morally relevant features may therefore require significant and highly convincing evidence that the scientific goals are well justified and that any benefits clearly outweigh the risks. In addition, for any model, researchers should consider whether similar benefits could be achieved with alternative approaches or a different experimental design that would result in fewer morally relevant features. In that case, in line with the least infringement principle, we would advocate that the model with the lesser concern is implemented if it can reasonably achieve the same objectives[20].

Additional responsibilities of researchers relate to how the research is conducted and how SCBEMs are used (see Table 1). Alongside this, there is a strong argument that regulation, governance and oversight should be applied to researchers by independent bodies or through legislation. Any system of governance should also consider who is performing the research, and what their motives and conflicts of interest might be. This is especially important for commercial and for-profit research, which is often carried out with less public visibility than work done in academic institutes or clinical settings. To maintain public trust in science, such research must be subject to open and transparent review. Regulators will therefore need to consider how best to implement policies that effectively regulate research in both academic and non-academic settings to ensure the field is governed responsibly and comprehensively.

While consensus has yet to be reached with regard to how the fast-paced nature of SCBEM research can be effectively regulated, in light of the international scope of scientific endeavour and the challenge of establishing suitable limits, it is clear that such regulation should be coherent, fair and transparent to promote both consistency and responsible research. To this end, an international consensus would enable a common ethical basis for research on an international level. We propose several criteria, building on the identified morally relevant features and responsibilities of researchers, that could be used as part of a holistic evaluation of SCBEM research proposals (Table 2).

We stress that drawing up any principles of regulation are not a one-off event, but should rather be considered an iterative process, embedded within the research programme (see [37]), and leading to proactive and continuous discussion between researchers, ethicists, lawyers, policy makers, regulators and the public, as these models and the field advance, in order to ensure research remains within acceptable boundaries.

## Conclusions and recommendations

In this Perspective, we have argued that regulation in the SCBEM field must continue to progress to ensure adequate research oversight, and safeguard public trust. In our view, biological features including the stage of development, the integration of organ systems, fetal potential and the capacity for forming neural circuitry, should be considered to determine what level of ethical oversight is warranted. However, none of these features alone can be used as a simplistic, stand-alone criterion for either allowing or preventing research avenues. All research oversight should be carefully balanced between the potential for beneficial impact versus its risks, and researchers must take accountability for the implications of their work within the wider ethical and social context. In addition, international coordination is important for progress in science, and a formal exchange of decision criteria and expert knowledge between SCROs may enable a more equitable and transparent decision-making process. We hope that these proposals might act as a launchpad for wider discussions around possible morally relevant features for ethical oversight, and how such oversight should be implemented, ultimately leading to greater clarity in the regulation of future SCBEM research.

## Figures and Tables

**Table 1:** Responsibilities of researchers and associated ethical and regulatory considerations

Responsibilities of Researchers	Ethical and Regulatory Considerations
**Accurately present work, conveying limitations of research and avoid over-reaching claims[38]. Scientific quality standards should also be applied[21].**	Important to promote public trust in science, particularly in the case of press releases and media reporting.
**Consider whether any biological and technical ‘work arounds’ might consequently raise other morally relevant features.**	Work arounds like using genetic ‘kill switches’ to prevent prolonged development^35^ or culturing advanced models in anaesthetic media to prevent sensory perception to intentionally reduce or remove this capacity (sometimes known as ‘disenhancement’[39, 40]) - while designed to avoid particular moral concerns - may simultaneously raise *different* questions, for example relating to the status of models whose capacities are merely suppressed rather than absent.
**Obtain all relevant consents for research.**	Critical when considering the stem cell-origins of SCBEMs, as has been discussed by others previously[5, 6]. While some cell lines may have broad consent for research purposes, it may be worthwhile considering deriving new lines with explicit consent for SCBEM creation.

**Table 2:** Morally relevant features to be considered as part of ethical oversight of SCBEM research, and related responsibilities of researchers.

	Features	Considerations
**Morally relevant features**	Developmental Stage	What is the developmental window that will be assessed with the SCBEM, and why? What is the set end point? (e.g. emergence of certain features, cell types or embryonic equivalent stages) With what frequency and methods will progression be monitored?
	Integration of Organ Systems	Which tissues or organ systems are likely to be present in the SCBEM? To what extent will these be integrated with one another? Are any emergent features likely to arise as a result of organ or tissue integration, and if so, which? Will any action be taken to minimise or prevent integration of organ systems, and if so, which?
	Fetal Potential	Does the SCBEM possess the intrinsic conditions to form a fetus? Is the SCBEM cultured or transplanted in such a way as to support elements that fulfil extraembryonic or uterine functions? If so, could these external conditions enable development towards fetal stages? Will any actions be taken to prevent prolonged development, and if so, which?
	Neuronal circuit-forming capacity	To what extent is the SCBEM likely to exhibit neuronal differentiation, neural function, and neural circuit-forming, and why (or why not)? Will any actions be taken to prevent the model developing mature neural circuit-forming capacity, and if so, which?
	Features relating to public concern	Is the SCBEM likely to exhibit any features that may be of public concern, and if so, which? Will any actions be taken to engage with the public ahead of, during and after the proposed research, and if so, which?
**Responsibilities of researchers**	What is the scientific justification for the research proposal? How will potential risks and harms be balanced against the potential for beneficial impact? Could alternative approaches or a different experimental design that would result in fewer morally relevant features be used? Have any conflicts of interest been identified and declared? What are the proposed downstream uses of the SCBEMs? Has relevant consent been obtained for all cell lines used during this project? How will scientific integrity be maintained during the research project?	
